# Female Oncofertility in Colorectal Cancer: Reproductive Uncertainties and Potential Benefits of Immunotherapy

**DOI:** 10.3390/jcm15145549

**Published:** 2026-07-15

**Authors:** Michele Miscia, Linda Cipriani, Nicole Conci, Tommaso Violante, Leonardo Notarangelo, Rossella Vicenti, Federica Cortese, Manuela Maletta, Marisol Doglioli, Antonio Raffone, Luigi Cobellis, Matteo Rottoli, Renato Seracchioli, Diego Raimondo

**Affiliations:** 1 Division of Gynaecology and Human Reproduction Physiopathology, IRCCS Azienda Ospedaliero-Universitaria di Bologna, 40138 Bologna, Italy; linda.cipriani@aosp.bo.it (L.C.); leonardo.notarangelo@unibo.it (L.N.); rossella.vicenti@unibo.it (R.V.); federica.cortese3@studio.unibo.it (F.C.); manuela.maletta2@unibo.it (M.M.); mdoglioli13@gmail.com (M.D.); renato.seracchioli@unibo.it (R.S.); diego.raimondo2@unibo.it (D.R.); 2 Department of Medical and Surgical Sciences (DIMEC), University of Bologna, 40126 Bologna, Italy; nicole.conci2@unibo.it (N.C.); tommaso.violante@unibo.it (T.V.); matteo.rottoli@unibo.it (M.R.); 3 Division of Medical Oncology, IRCCS Azienda Ospedaliero-Universitaria di Bologna, 40138 Bologna, Italy; 4 Surgery of the Alimentary Tract, IRCCS Azienda Ospedaliero-Universitaria di Bologna, 40138 Bologna, Italy; 5 Division of Colon and Rectal Surgery, Mayo Clinic, Rochester, MN 55905, USA; 6 Department of Woman, Child, and General and Specialized Surgery, University of Campania “Luigi Vanvitelli”, 80138 Naples, Italy; luigi.cobellis@unicampania.it

**Keywords:** colorectal cancer, immunotherapy, immune checkpoint inhibitors, oncofertility, fertility preservation, ovarian reserve, reproductive health, oocyte cryopreservation, ovarian tissue cryopreservation

## Abstract

Early-onset colorectal cancer is increasing, making reproductive health an increasingly relevant survivorship issue. While immune checkpoint inhibitors have altered the management of deficient mismatch repair/microsatellite instability-high (dMMR/MSI-H) colorectal cancer, their implications for reproductive-age women remain insufficiently defined. We performed a focused narrative review, structured in line with the SANRA framework, to clarify this specific clinical domain. Immunotherapy may reshape female oncofertility counseling through two distinct mechanisms: direct reproductive uncertainty, including established endocrine toxicities and hypothesized, but currently unproven, direct gonadal effects; and indirect pathway-modifying effects, such as the potential avoidance of pelvic radiotherapy or radical surgery in selected patients with locally advanced dMMR/MSI-H rectal cancer. Anatomical organ preservation should not be automatically equated with functional fertility preservation. The key clinical message is that reproductive counseling should not be restricted to historically gonadotoxic chemotherapy. Instead, early multidisciplinary guidance should explicitly distinguish CRC-specific evidence from extrapolated or theoretical concerns, integrate fertility preservation strategies when clinically feasible, and address pelvic and sexual health, contraception, washout, endocrine follow-up, and future pregnancy planning. Until prospective CRC-specific reproductive data become available, immunotherapy should not be presented as either reproductively neutral or fertility-preserving.

## 1. Introduction

Colorectal cancer (CRC) remains one of the most common malignancies worldwide and a major cause of cancer mortality [1]. Although traditionally regarded as a disease of later adulthood, the incidence of early-onset CRC (EOCRC), usually defined as diagnosis before 50 years of age, has increased in multiple populations [2,3]. Recent population-level analyses confirm that this trend is clinically relevant for women diagnosed during their reproductive years, including those in their twenties, thirties and early forties [4,5]. The definition of “reproductive-age” varies across oncology, reproductive medicine and epidemiological research. While EOCRC is conventionally defined by an age at diagnosis below 50 years, reproductive medicine often relies on narrower or individualized chronological limits reflecting the physiological decline in ovarian reserve. Therefore, throughout this review, “reproductive-age” is used as a broad clinical concept rather than as a rigid chronological cutoff, emphasizing the need for individualized assessment. This epidemiologic shift matters because CRC treatment can affect reproductive health through several pathways: systemic therapy may delay attempts at conception or impair ovarian function; pelvic radiotherapy can damage ovarian and uterine function; rectal surgery can alter pelvic anatomy, autonomic innervation and sexual function; and prolonged treatment or surveillance can consume reproductive time even when ovarian reserve is not directly depleted [6,7,8,9]. Although immune checkpoint inhibitors may also raise reproductive and endocrine concerns in men, the present review is deliberately focused on female reproductive health. This focus reflects the time-sensitive nature of ovarian stimulation and fertility preservation procedures, as well as the specific implications of pelvic treatment for uterine function, pregnancy feasibility and long-term pelvic survivorship [10,11,12]. In CRC, reproductive risk extends beyond a simple estimate of ovarian reserve. Rectal cancer can expose women to ovarian insufficiency, uterine radiation, dyspareunia, altered pelvic anatomy, bowel and urinary dysfunction, stoma-related body-image concerns and uncertainty about future pregnancy feasibility [7,8,9]. In colon cancer, reproductive counseling is generally less related to pelvic functional damage and more to systemic treatment, treatment-related delay and the timing of future pregnancy attempts [6,7,8,9]. Contemporary fertility preservation guidance recommends early counseling and referral for post-pubertal patients at risk of treatment-related infertility or reproductive compromise; however, implementation remains inconsistent when planned treatment is not classically considered highly gonadotoxic [10,11,12]. Immune checkpoint inhibitors (ICIs) add a distinct layer of complexity. Their established role in CRC is concentrated in tumors with deficient mismatch repair or high microsatellite instability (dMMR/MSI-H), a biologically distinct subgroup characterized by high neoantigen load and marked sensitivity to PD-1-based therapy [13]. In metastatic dMMR/MSI-H CRC, KEYNOTE-177 established pembrolizumab as a first-line standard, while CheckMate 8HW supports nivolumab plus ipilimumab as another immune-based option in unresectable or metastatic dMMR/MSI-H disease [14,15,16,17]. Contemporary guidelines for rectal and metastatic CRC now explicitly incorporate immunotherapy in selected dMMR/MSI-H settings, with treatment decisions adapted to disease stage, tumor site and molecular profile [18,19,20,21,22]. In non-metastatic disease, neoadjuvant and perioperative immunotherapy strategies have produced high pathological and clinical response rates in selected dMMR/MSI-H colon and rectal cancer cohorts, but their implementation remains jurisdiction-, protocol- and pathway-dependent rather than universally approved in routine practice [23,24,25,26,27,28]. The clinical question is therefore not whether immunotherapy should be described as reproductively neutral or gonadotoxic. In CRC, the more useful question is how immunotherapy may modify the overall reproductive burden of treatment. This includes direct uncertainty regarding ovarian reserve, menstrual function, endocrine toxicity, pregnancy timing, contraception, washout and lactation, as well as indirect effects mediated through treatment-pathway modification. These indirect effects are most evident in dMMR/MSI-H rectal cancer, where selected patients who achieve and maintain a complete clinical response may avoid or defer pelvic radiotherapy and radical surgery. However, anatomical organ preservation should not be equated with established preservation of reproductive function. Direct CRC-specific reproductive data after ICI exposure are currently lacking [29,30]. Accordingly, the proposed counseling framework is informed by indirect CRC treatment–pathway evidence [23,24,25,26,27,28], evidence extrapolated from other solid tumors [31,32,33,34], established endocrine toxicity profiles [35,36], and biologically plausible mechanisms [37,38], rather than by robust CRC-specific reproductive endpoints [39]. This review synthesizes the available evidence to support clinically usable, pathway-based fertility preservation and reproductive-health counseling for women of reproductive age with CRC considered for immune checkpoint blockade.

## 2. Materials and Methods

**Figure 1:**
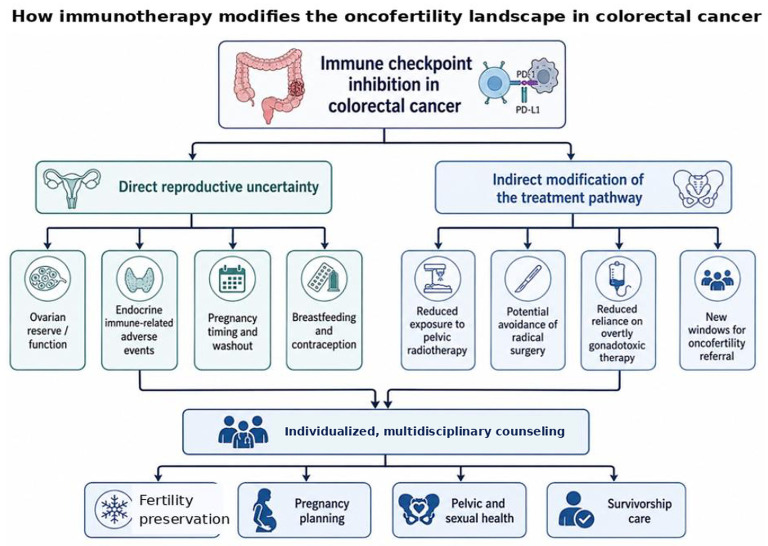
Conceptual framework of oncofertility in colorectal cancer treated with immune checkpoint inhibitors. Immune checkpoint inhibition may influence reproductive counseling through two complementary dimensions. Direct reproductive uncertainty includes possible effects on ovarian reserve and function, menstrual pattern, endocrine immune-related adverse events, pregnancy timing, contraception, washout and breastfeeding. Indirect pathway modification includes the possibility, in selected dMMR/MSI-H settings, of reducing exposure to pelvic radiotherapy, radical pelvic surgery or more burdensome systemic treatment. These dimensions converge into individualized multidisciplinary counseling addressing fertility preservation, pregnancy planning, pelvic and sexual health, hereditary cancer considerations and survivorship care.

**Figure 2:**
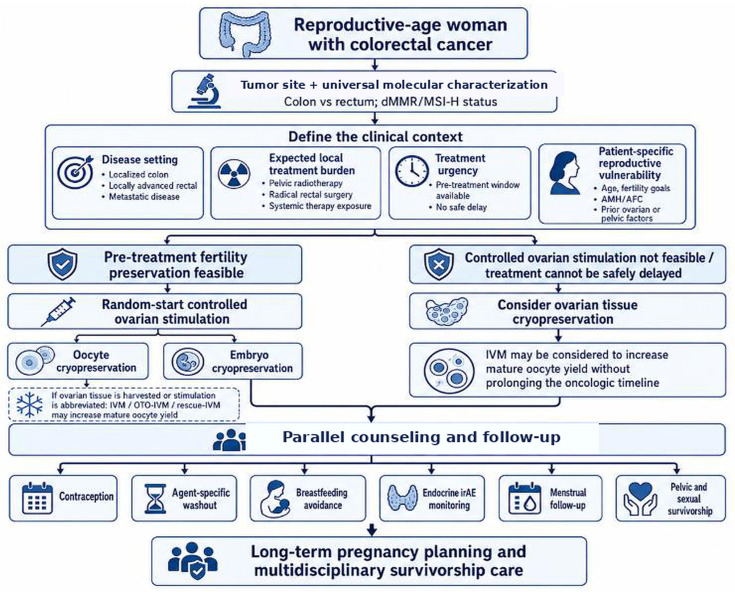
Proposed oncofertility pathway for reproductive-age women with dMMR/MSI-H colorectal cancer. The pathway begins with tumor-site definition and molecular characterization, because dMMR/MSI-H status identifies the subgroup in which immune checkpoint blockade may alter the oncologic pathway. Reproductive counseling should integrate disease setting, expected local treatment burden, treatment urgency and patient-specific reproductive vulnerability. Fertility preservation options should be selected according to the available oncologic window, while contraception, agent-specific washout, breastfeeding avoidance, endocrine immune-related adverse event monitoring, menstrual follow-up, pelvic and sexual survivorship, and long-term pregnancy planning should proceed in parallel rather than being deferred to survivorship.

This focused narrative review was structured with reference to the SANRA scale for narrative reviews to improve transparency and methodological consistency [40]. PubMed/MEDLINE was searched using six complementary strategies addressing: colorectal cancer treatment pathways and molecular stratification; neoadjuvant immunotherapy in dMMR/MSI-H colon cancer; immunotherapy and organ-preservation strategies in dMMR/MSI-H rectal cancer; immune checkpoint inhibitor-related reproductive and endocrine effects; pregnancy and lactation during or after immune checkpoint inhibition; and reproductive, pelvic and sexual consequences of colorectal cancer treatment. Representative search combinations included (“colorectal cancer” OR “colon cancer” OR “rectal cancer”) AND (“mismatch repair deficiency” OR “dMMR” OR “microsatellite instability” OR “MSI-H”) AND (“immune checkpoint inhibitor*” OR “pembrolizumab” OR “nivolumab” OR “ipilimumab” OR “dostarlimab”); immune checkpoint inhibitor terms combined with “fertility preservation”, “oncofertility”, “ovarian reserve”, “anti-Müllerian hormone”, “AMH”, “antral follicle count”, “menstrual function”, “hypophysitis”, “thyroiditis”, “pregnancy”, “breastfeeding” or “lactation”; and colorectal cancer terms combined with “fertility”, “reproductive health”, “sexual function”, “pelvic radiotherapy”, “uterine radiation”, “ovarian transposition” or “rectal surgery”. No lower publication-date limit was applied; records published up to 8 May 2026 and indexed in English were considered. The six database searches initially identified 2373 records. Fifty-four records published after the predefined cutoff were removed, leaving 2319 records; after deduplication, 2180 unique titles and abstracts were screened for relevance to the review question. Full texts were evaluated when necessary to assess eligibility or extract clinically relevant information. International oncology and reproductive-medicine guidelines [10,11,12,18,19,20], colorectal cancer clinical practice guidelines [21,22], and U.S. Food and Drug Administration prescribing information for pembrolizumab, nivolumab, ipilimumab and dostarlimab [41,42,43,44] were identified through targeted searches of official sources. Eligible publications addressed early-onset colorectal cancer, dMMR/MSI-H treatment pathways, immune-related endocrine adverse events, fertility preservation, reproductive or sexual health, pregnancy, lactation, or reproductive safety during or after immune checkpoint inhibition. Case reports with limited generalizability and studies focused exclusively on non-reproductive survivorship issues were not routinely included. Final reference selection was based on clinical relevance, methodological quality, recency, and direct applicability to the predefined clinical domains. CRC-specific evidence was prioritized for epidemiology, molecular stratification, treatment sequence, organ preservation and survivorship implications. When direct CRC-specific reproductive data were unavailable, evidence from melanoma and mixed-tumor cohorts, preclinical ovarian models, endocrine toxicity literature, pregnancy pharmacovigilance and tumor-specific reviews was included only when clinically relevant and was explicitly considered extrapolative [29,30,31,32,33,34,35,36,37,38,45,46]. Phase II and III trials, clinical practice guidelines, regulatory labels and systematic reviews were considered high-priority evidence. No formal risk-of-bias assessment or quantitative pooling was undertaken because the objective was a clinically oriented narrative synthesis rather than a systematic review. The hierarchy of evidence and its direct applicability to colorectal cancer are summarized in Table 1. Figure 1 and Figure 2 are author-conceived conceptual illustrations generated with the assistance of ChatGPT Images 2.0 (OpenAI, San Francisco, CA, USA). The authors reviewed the final figures for scientific accuracy and consistency with the cited literature and take full responsibility for their content.

## 3. Colorectal Cancer Treatment Pathways

Colorectal cancer does not represent a uniform oncofertility scenario. The reproductive implications of treatment differ between colon and rectal cancer because exposure to pelvic radiotherapy, radical pelvic surgery and multimodality treatment is largely determined by tumor site, stage and treatment strategy. In colon cancer, management is primarily defined by pathological T and N stage, resectability, emergency presentation, risk features and MMR/MSI status. In rectal cancer, management is additionally determined by pelvic MRI risk stratification, distance from the anal verge, mesorectal fascia involvement, sphincter preservation, nodal burden and the anticipated need for neoadjuvant therapy. These distinctions are clinically relevant because pelvic radiotherapy and radical rectal surgery may affect uterine capacity, ovarian reserve, pelvic autonomic function, bowel function, sexual health and pregnancy feasibility in ways that colectomy alone generally does not [7,8,9,18,19,21,22]. For colon cancer, stage 0 and selected low-risk T1 lesions may be managed endoscopically if resection is complete and adverse pathological features are absent; T1 lesions with high-risk features and most stage I–III cancers require oncologic colectomy with adequate lymph-node assessment. Stage I disease is generally treated with surgery alone. Stage II disease is managed after resection according to high-risk pathological features and MMR/MSI status; adjuvant fluoropyrimidine monotherapy is generally not favored for dMMR/MSI-H stage II tumors, whereas selected high-risk pMMR/MSS patients may be considered for adjuvant chemotherapy. Stage III colon cancer usually requires postoperative oxaliplatin-based adjuvant chemotherapy, with duration and regimen adapted to recurrence risk and tolerance. In non-metastatic dMMR/MSI-H colon cancer, neoadjuvant immune checkpoint blockade has produced high pathological response rates in NICHE-2 and subsequent datasets. However, it remains a trial-, pathway- and jurisdiction-dependent strategy rather than a universally approved replacement for standard surgery-based management [21,23,24,25]. For rectal cancer, early cT1N0 tumors with favorable histological criteria may be considered for local excision, whereas deeper or node-positive tumors are usually managed with total mesorectal excision within a risk-adapted multimodality pathway. In locally advanced rectal cancer, baseline high-resolution pelvic MRI and pretreatment MMR/MSI testing are essential because treatment may include long-course chemoradiotherapy, short-course radiotherapy, total neoadjuvant therapy, surgery, or nonoperative management after complete clinical response. For dMMR/MSI-H locally advanced rectal cancer, immunotherapy is a guideline-supported treatment approach. ASCO recommends immunotherapy for MSI-H/dMMR tumors, while ESMO guidance includes planned dostarlimab therapy, with strict response assessment and surveillance [18,19,22,26,27,28]. This is the setting in which immunotherapy has the clearest indirect oncofertility relevance, because complete clinical response may allow selected patients to avoid pelvic radiotherapy and radical rectal surgery, although organ preservation must not be equated with proven reproductive safety. In metastatic CRC, treatment is defined by resectability of metastases, disease tempo, prior therapies, sidedness, RAS/BRAF status, HER2 status, where relevant, and MMR/MSI status. For dMMR/MSI-H metastatic CRC without contraindications to immunotherapy, pembrolizumab and nivolumab plus ipilimumab are established first-line immune-based options; treatment selection should be adapted to the relevant regulatory and guideline context, prior treatment exposure, disease characteristics and patient factors [14,15,16,17,20]. These regimens are not fertility preservation interventions, but durable disease control can create long treatment horizons during which contraception, endocrine toxicity, treatment discontinuation, washout and future pregnancy planning become clinically relevant for selected long-term responders. Molecular status is therefore not only a predictive biomarker for treatment selection but also a practical trigger for reproductive assessment. dMMR/MSI-H CRC is associated with increased neoantigenicity and sensitivity to checkpoint blockade [13,57]. Its prevalence varies by stage, age and tumor site, and it is generally higher in early-stage disease than in unselected metastatic CRC [57]. In young patients, dMMR/MSI-H status should also raise the possibility of Lynch syndrome, with implications for cancer surveillance, family counseling and reproductive planning, including possible future preimplantation genetic testing when a pathogenic familial variant is identified [58]. In reproductive-age women, confirmation of dMMR/MSI-H disease should therefore prompt not only therapeutic stratification but also timely documentation of reproductive goals and baseline reproductive vulnerability when future pregnancy is relevant. Consequently, the integration of immunotherapy into these specific disease settings may modify the overall clinical approach to reproductive counseling. Figure 1 summarizes the dual nature of this evolving counseling framework.

## 4. Reproductive Biology of Checkpoint Blockade

The reproductive rationale for concern under immune checkpoint blockade differs from the logic of classical chemotherapy-induced gonadotoxicity. Alkylating agents and pelvic radiotherapy can damage the ovary through relatively direct follicular depletion, stromal injury or vascular injury. ICIs do not primarily act through these mechanisms. Their potential reproductive effects are more plausibly mediated by immune dysregulation, inflammatory signaling within the ovarian microenvironment, endocrine organ dysfunction and interference with maternal–fetal immune tolerance [29,30,31,32,37,38,45]. This distinction is clinically important: absence of classical cytotoxicity does not prove reproductive neutrality, but biological plausibility does not allow deterministic prediction of infertility. Preclinical evidence supports concern but remains indirect. Murine models of PD-1/PD-L1 or CTLA-4 blockade have shown intra-ovarian immune activation, T-cell infiltration, cytokine signaling, follicular loss and impaired oocyte competence [37,38]. These findings suggest that checkpoint pathways may contribute to ovarian immune homeostasis and that pharmacologic blockade could alter follicular survival or oocyte quality in susceptible contexts. Translation to reproductive-age women with CRC is limited by species differences, age at exposure, dosing, tumor context, combination therapy and the absence of CRC-specific prospective reproductive cohorts. Preclinical data should therefore be used to justify early counseling and structured monitoring, not to provide numerical estimates of ovarian failure. Beyond static ovarian reserve, checkpoint disruption may also affect functional reproductive processes such as follicular growth, ovulation and luteal maintenance, because immune effector cells contribute physiologically to these events. This further supports longitudinal assessment of menstrual pattern and endocrine function alongside AMH and AFC [32]. Human data remain sparse and mostly non-CRC-specific. Systematic reviews on ICIs, fertility, pregnancy and sexual health emphasize the paucity of direct reproductive endpoints [29,30]. Similar considerations apply to other ICI-treated tumor settings, including melanoma and renal cell carcinoma, in which label-based contraception, washout and lactation requirements coexist with unresolved questions on ovarian reserve, endocrine dysfunction and post-treatment pregnancy safety [31,32]. Recent melanoma data provide mixed but clinically relevant signals: one cohort suggested generally reassuring short-term menstrual outcomes, whereas analyses of ovarian reserve markers in young women treated with ipilimumab-based therapy reported declines in anti-Müllerian hormone, although generalizability to PD-1-based CRC regimens is limited [33,34]. To distinguish established evidence from theoretical concerns, four potential mechanisms of reproductive impairment should be considered separately. First, endocrine-mediated reproductive dysfunction is an established and clinically actionable risk, usually resulting from secondary anovulation caused by immune-related endocrine adverse events such as hypophysitis or thyroid dysfunction. Second, treatment-related delay in conception is a practical risk, because active treatment, the need to avoid pregnancy during therapy and agent-specific post-treatment intervals may reduce reproductive time independently of direct drug toxicity. In contrast, impaired ovarian reserve, defined as a subclinical decline in the follicle pool reflected by lower AMH or AFC without overt menstrual cessation, and primary ovarian insufficiency, defined as hypergonadotropic hypogonadism with premature menopause, remain unproven direct effects supported only by biological plausibility and sparse non-CRC human data. Distinguishing these entities avoids conflating established endocrine or time-related risks with unproven direct gonadal damage. Endocrine immune-related adverse events are the most actionable reproductive mechanism. Thyroid dysfunction is common during PD-1/PD-L1 blockade, whereas hypophysitis and broader pituitary dysfunction are more characteristic of CTLA-4-containing regimens and combination strategies [35,36]. These events can impair fertility even when ovarian follicle number is preserved. Hypothyroidism can disturb ovulation and complicate pregnancy; hypophysitis can cause hypogonadotropic hypogonadism, amenorrhea, adrenal insufficiency or long-term hormone replacement needs; and fatigue, menstrual changes or sexual symptoms may be misattributed to cancer or treatment burden unless endocrine assessment is actively pursued [35,36]. In reproductive-age women treated with ICIs, endocrine follow-up is therefore not only toxicity surveillance; it is part of reproductive care. Pregnancy requires a separate mechanistic and regulatory discussion. Maternal–fetal tolerance depends on regulated immune signaling at the decidual–placental interface, where PD-1/PD-L1 and CTLA-4 pathways contribute to limiting maternal effector responses against fetal antigens [29,46]. Blocking these pathways during pregnancy raises plausible concerns about implantation, miscarriage, placental dysfunction, fetal growth and neonatal immune effects. Human evidence remains limited and heterogeneous, with pharmacovigilance data unable to provide CRC-specific risk estimates [46]. The appropriate clinical posture is therefore conservative: pregnancy should be avoided during active ICI therapy and deferred until the relevant label-based post-treatment interval has elapsed, with individualized oncologic reassessment before conception is attempted [41,42,43,44].

## 5. Fertility Preservation and Counseling

Fertility preservation counseling in CRC should be pathway-based rather than drug-label-based alone. The first step is to define tumor site, stage, molecular subtype, treatment urgency, expected local treatment burden, planned systemic therapy and the patient’s reproductive goals. A 35-year-old woman with localized dMMR/MSI-H colon cancer considered for neoadjuvant immunotherapy has a different counseling window from a woman with dMMR/MSI-H locally advanced rectal cancer in whom organ preservation is being discussed, and both differ from a woman with metastatic disease starting urgent PD-1-based therapy. The common principle is that reproductive goals should be documented before treatment decisions become irreversible. Pretreatment fertility counseling is clinically meaningful even when the magnitude of treatment-related reproductive risk is uncertain, because it improves preparedness, supports reproductive autonomy and can reduce subsequent decisional regret [48,49]. ASCO, ESMO and ESHRE guidance support early counseling and timely referral to reproductive specialists for patients interested in or uncertain about fertility preservation [10,11,12]. This point is especially relevant for ICI-treated CRC: the patient may ultimately decline cryopreservation, or oncologic urgency may preclude intervention, but the decision should be informed and documented rather than omitted because immunotherapy is not a classical gonadotoxic exposure. Baseline reproductive assessment should be explicit. It should include age, parity, desire for future pregnancy, menstrual history, prior infertility, previous ovarian or pelvic surgery, endometriosis or ovarian cysts, hormonal contraception, prior chemotherapy or radiotherapy, planned pelvic treatment, family history suggestive of hereditary cancer and the expected oncologic timeline. AMH and antral follicle count can contextualize baseline ovarian reserve and likely response to stimulation, although they do not predict spontaneous pregnancy with certainty and have not been validated as predictors of ICI-related gonadal injury [50,51,52]. Endometriosis and endometriomas should also be documented, because endometriotic cysts may reflect an additional baseline ovarian vulnerability and have been associated with apoptotic changes in adjacent ovarian cortex [53]. AMH and AFC have pragmatic value because they help convert an abstract risk discussion into an individualized estimate of baseline reproductive vulnerability and expected oocyte yield. Mature oocyte cryopreservation and embryo cryopreservation remain the most established fertility preservation options for post-pubertal women when controlled ovarian stimulation can be performed without compromising oncologic care [10,11,12,54,55]. Modern antagonist and random-start protocols allow initiation irrespective of cycle phase and usually require approximately 10–14 days from stimulation start to oocyte retrieval [10,11,12]. In patients with suspected or confirmed Lynch syndrome, genetic counseling is an integral component of the pathway. Identification of a pathogenic germline variant has implications for cascade testing, reproductive autonomy and future family-building decisions. Oocyte cryopreservation avoids the need for sperm at diagnosis and preserves autonomy for women without a partner or who do not wish to create embryos. Embryo cryopreservation requires fertilization before freezing but may facilitate future PGT-M when a validated familial-variant testing pathway is available. These options should be discussed according to the patient’s preferences, clinical timeline and long-term reproductive plans [58]. Stimulation protocols should minimize procedural risk without overstating hormone sensitivity. CRC is not generally treated as an estrogen-driven malignancy in the way some breast cancers are. The main practical questions are whether a brief stimulation interval is oncologically acceptable, whether the patient’s surgical or thrombotic risk profile allows stimulation and retrieval, and whether pelvic symptoms or obstruction impose urgency. Antagonist protocols with individualized gonadotropin dosing and GnRH-agonist trigger can reduce ovarian hyperstimulation risk in high responders. Letrozole-supplemented stimulation can be considered when estradiol minimization is clinically desired, but routine use in CRC is not supported by tumor-specific evidence [10,11,12]. Counseling should be concrete: stimulation requires injections, ultrasound and biochemical monitoring, transvaginal oocyte retrieval and coordination with oncology to preserve the planned treatment start date. Ovarian tissue cryopreservation is a complementary option when stimulation is infeasible, treatment cannot be delayed or the patient wishes to preserve future reproductive and endocrine options [10,11,12,56]. It avoids ovarian stimulation but requires laparoscopic retrieval of ovarian cortex and later reimplantation if the tissue is to be used. In CRC, the risk of ovarian contamination is not analogous to that in hematologic malignancies, but direct or metastatic ovarian involvement must be considered in advanced disease and discussed with oncology before tissue use. In vitro maturation may be considered in highly selected time-sensitive pathways, including ovarian tissue oocyte in vitro maturation when immature oocytes are obtained during ovarian tissue cryopreservation or rescue in vitro maturation after abbreviated stimulation. Outcomes remain less consistent and less widely available than conventional mature oocyte or embryo cryopreservation, so IVM should be presented as an adjunctive yield strategy rather than a substitute for established options [10,11,12]. To operationalize these options, clinical decision-making should follow a concise pathway defined by oncologic urgency and anticipated local therapies. When a 10–14-day stimulation window is available, random-start controlled ovarian stimulation for oocyte or embryo cryopreservation remains the established option [10,11,12]. When treatment cannot safely be delayed, including selected patients with metastatic disease requiring immediate systemic therapy, ovarian tissue cryopreservation, with or without in vitro maturation of retrieved immature oocytes, may be considered, provided that the surgical procedure is feasible and does not delay urgent oncologic care [10,11,12,56]. When pelvic radiotherapy is planned, ovarian transposition should be discussed and considered in appropriately selected patients before irradiation. It may reduce ovarian radiation exposure but does not protect uterine function and does not replace cryopreservation or counseling regarding future obstetric risks [10,11,12]. If radical rectal surgery is planned, counseling should also encompass the risk of autonomic nerve damage and altered pelvic anatomy [7,8,9]. In young patients with suspected or confirmed Lynch syndrome, embryo cryopreservation may be specifically discussed when future PGT-M is being considered [58]. Automatic referral triggers can reduce missed counseling. Reproductive age, documented desire for future pregnancy, dMMR/MSI-H status with planned immunotherapy, anticipated pelvic radiotherapy, rectal tumor location, possible organ-preservation strategy and a clinically acceptable treatment interval should prompt referral before systemic or pelvic therapy begins. The primary objective is not to recommend cryopreservation universally, but to ensure that the decision to proceed or not proceed is informed, documented and made before the relevant therapeutic window closes. Representative clinical scenarios and related counseling priorities are summarized in Table 2.

## 6. Pregnancy, Washout and Endocrine Follow-Up

Contraception, washout and lactation counseling should be handled separately from the decision to cryopreserve. A woman may decline fertility preservation but still need precise pregnancy-avoidance counseling; conversely, a woman may undergo oocyte or embryo cryopreservation but still require a future transfer plan after treatment completion, drug washout and oncologic reassessment. For the ICIs most relevant to CRC, regulatory product information supports effective contraception during therapy and for a defined interval after the last dose. Based specifically on U.S. Food and Drug Administration (FDA) prescribing information, the post-treatment intervals are 4 months for pembrolizumab, 5 months for nivolumab, 3 months for ipilimumab, and 4 months for dostarlimab [41,42,43,44]. Under FDA labeling, breastfeeding is discouraged during treatment and for the same agent-specific post-treatment intervals [41,42,43,44]. These intervals reflect FDA product information and do not imply universal global applicability; the current product information in the relevant jurisdiction should be verified before individual counseling. In combination regimens, the longest relevant interval should generally be applied. Key FDA-label-informed reproductive safety counseling points are summarized in Table 3.

Localized dMMR/MSI-H colon cancer offers a defined opportunity for anticipatory counseling because diagnosis, molecular status, surgical planning and systemic treatment decisions often converge within a short interval. If neoadjuvant immunotherapy is considered, reproductive goals and fertility preservation feasibility should be assessed before treatment initiation. If upfront surgery is planned, counseling may occur postoperatively before adjuvant decisions are finalized. dMMR/MSI-H status should therefore not be used only to select systemic therapy; in reproductive-age women, it should also prompt documentation of fertility intentions and baseline ovarian reserve when future pregnancy is relevant.

In locally advanced rectal cancer, reproductive counseling should account for both gonadal and non-gonadal consequences of treatment. Traditional reproductive harm is not limited to ovarian reserve: pelvic radiotherapy may damage ovarian and uterine function, total mesorectal excision can affect autonomic nerves and sexual function, and stoma formation can influence intimacy and pregnancy planning [7,8,9,18,19]. In dMMR/MSI-H rectal cancer, immunotherapy introduces the possibility of organ preservation after complete clinical response, but this should not be translated into blanket reassurance. Nonoperative management requires intensive surveillance, incomplete response remains possible, and long-term obstetric, sexual and pelvic-floor outcomes after immune-based organ preservation remain incompletely characterized [26,27,28]. Counseling should therefore address both the potential benefit of avoiding pelvic-damaging therapy and the uncertainty that remains after favorable oncologic response.

In metastatic dMMR/MSI-H CRC, counseling is usually less about immediate cryopreservation for all patients and more about maintaining reproductive clarity during prolonged treatment. Some women require urgent therapy, making stimulation inappropriate. Others may have indolent disease, excellent response or a treatment interval in which fertility preservation could be considered. In all cases, contraception must be addressed before treatment begins. For durable responders, especially after prolonged disease control on PD-1-based therapy, including after approximately two years in regimens using fixed-duration immunotherapy, oncology teams may consider treatment interruption and surveillance in selected cases, potentially creating a treatment-free interval in which reproductive questions become more concrete [14,17,20]. Future pregnancy questions should still be revisited only after oncologic reassessment, confirmation of an adequate drug-free interval, endocrine stability and multidisciplinary evaluation, including maternal–fetal medicine when pregnancy is contemplated.

Longitudinal follow-up should include menstrual and endocrine assessment rather than ovarian reserve alone. Amenorrhea during or after ICI treatment may reflect pregnancy, hormonal contraception, hypothalamic suppression, weight change, stress, thyroid dysfunction, hypophysitis, primary ovarian insufficiency or treatment-related systemic illness. These diagnoses carry different implications and should not be conflated. A pragmatic follow-up strategy should document menstrual pattern, symptoms suggestive of endocrine irAEs, thyroid function, pituitary evaluation when clinically indicated and AMH/AFC when future fertility or fertility preservation remains relevant [35,36,50,51,52]. This is particularly important in metastatic disease, where treatment may continue for years, and in rectal cancer survivors, where pelvic and sexual function may remain central determinants of reproductive quality of life. 

A pathway-based framework for oncofertility counseling in reproductive-age women with dMMR/MSI-H CRC is summarized in Figure 2.

## 7. Clinical Implementation and Research Priorities

Several interpretive cautions should frame clinical implementation. First, direct CRC-specific reproductive evidence under immunotherapy is almost absent. No prospective CRC cohort currently defines how AMH, AFC, menstrual recovery, primary ovarian insufficiency, fertility preservation outcomes or post-treatment pregnancy evolve after PD-1 or CTLA-4 blockade. Counseling therefore relies on extrapolation from melanoma, mixed-tumor ICI literature, endocrine toxicity data, pregnancy pharmacovigilance and preclinical models [29,30,31,32,33,34,35,36,37,38,45,46]. This extrapolation is reasonable only if its limits are stated explicitly. Current evidence supports counseling, monitoring and prospective data collection; it does not support precise CRC-specific risk prediction.

Second, the indirect reproductive implications of immunotherapy should be neither minimized nor overstated. In dMMR/MSI-H rectal cancer, avoidance of pelvic radiotherapy or radical surgery may be highly relevant to fertility, uterine function, pelvic anatomy, sexual function and quality of life [18,19,26,27,28]. However, organ preservation after complete response is a monitored oncologic strategy, not a guarantee of reproductive safety. Conversely, when pelvic radiotherapy or radical surgery remains necessary, counseling should not stop at oocyte cryopreservation. It should also address uterine exposure, pelvic functional outcomes, sexual health, obstetric feasibility and the psychological effects of altered body image or stoma formation [7,8,9].

Third, oncology and reproductive medicine teams need shared operational language. For oncologists, fertility preservation should be described in concrete terms: who needs referral, how long stimulation usually takes, what procedures are involved, and when treatment should not be delayed. For reproductive specialists, CRC should be described by treatment pathway rather than tumor label alone: colon versus rectum, localized versus metastatic disease, dMMR/MSI-H versus pMMR/MSS biology, expected radiotherapy, surgical plan, systemic regimen and treatment duration. Miscommunication at this interface can produce two opposite errors: ICIs may be ignored because they are not considered gonadotoxic, while immune-based organ preservation may be overinterpreted as complete reproductive protection. Both forms of misclassification may compromise balanced counseling.

Experience from tumor-specific ICI oncofertility frameworks also indicates that access, reimbursement, local service capacity and absence of standardized referral triggers may determine whether counseling occurs in time, even when the clinical rationale is recognized [31,32].

Prospective CRC immunotherapy cohorts should prioritize, first, longitudinal characterization of ovarian and menstrual function alongside endocrine immune-related adverse events; second, fertility preservation feasibility and outcomes, including referral, uptake, oocyte yield and treatment delay; and third, post-treatment pregnancy, uterine, pelvic, sexual and psychosocial outcomes. Minimal datasets should include age, parity, fertility intentions, menstrual history, hormonal contraception, AMH, AFC, FSH/LH/estradiol when appropriate, thyroid function, pituitary toxicity, endocrine replacement, fertility preservation referral, cryopreservation uptake, oocyte yield, treatment delay, pregnancy attempts, miscarriage, live birth and obstetric outcomes. These variables should be collected at baseline, during treatment, after treatment and at defined survivorship intervals. Reproductive endpoints should also be embedded in neoadjuvant dMMR/MSI-H colon and rectal cancer platforms, where molecular selection, prospective follow-up and multidisciplinary planning already exist [23,24,25,26,27,28].

Future studies should distinguish ovarian, endocrine, uterine, pelvic, sexual and psychosocial endpoints rather than treating fertility as a single outcome. Systematic reviews of ICI-treated CRC appropriately focus on response, survival and toxicity, but reproductive endpoints remain largely absent [59,60,61,62,63,64]. Sex-specific analyses should also be expanded because immune response, toxicity, CRC biology and survivorship concerns may differ between women and men [65,66,67,68]. Recent calls to include reproductive health outcomes in standard toxicity assessment and oncology trials support this priority, and emerging mixed-tumor hormone data, pharmacovigilance analyses and broader reviews of immunotherapy and small-molecule therapy further emphasize the need for prospective, treatment-specific reproductive monitoring [39,47,69,70,71]. In reproductive-age women, the relevant question is not only whether AMH declines after ICI exposure. It is whether immunotherapy-containing pathways preserve, impair or reconfigure the conditions under which pregnancy and reproductive autonomy remain possible after CRC treatment.

## 8. Conclusions

In CRC, immunotherapy primarily reshapes reproductive counseling by changing anticipated treatment exposure; its long-term reproductive safety profile remains incompletely characterized. In dMMR/MSI-H metastatic CRC, PD-1-based therapy can extend survival and make reproductive planning relevant for selected long-term responders. In localized colon cancer, neoadjuvant or perioperative immunotherapy may alter systemic treatment sequence in specialized pathways. In locally advanced dMMR/MSI-H rectal cancer, PD-1 blockade may reduce exposure to pelvic radiotherapy or radical surgery in selected complete responders, with potentially major implications for fertility, pelvic function, sexual health and future pregnancy.

At the same time, the direct reproductive effects of ICIs on ovarian reserve, menstrual function, endocrine health, pregnancy timing and long-term reproductive outcomes remain insufficiently defined. Fertility preservation counseling should therefore not be restricted to therapies already classified as highly gonadotoxic. The appropriate approach is early, individualized, pathway-based and multidisciplinary counseling that distinguishes evidence-secure recommendations from extrapolative uncertainty. Until CRC-specific reproductive data are available, immunotherapy may reduce exposure to established reproductive harms in selected treatment pathways, but it should not be presented as either reproductively neutral or fertility-preserving. High-quality care depends on timely referral, baseline reproductive assessment, realistic discussion of fertility preservation options and timelines, agent-specific contraception and washout planning, endocrine monitoring and prospective research embedded within CRC immunotherapy pathways.

## Figures and Tables

**Table 1:** Hierarchy and classification of evidence domains informing female oncofertility counseling in immunotherapy-treated colorectal cancer.

Evidence Domain	Clinical Relevance and Limitations in Colorectal Cancer	Principal Literature Base
Direct CRC-specific reproductive evidence	No prospective CRC cohort reporting longitudinal AMH, AFC, menstrual function, fertility-preservation outcomes or post-treatment pregnancy outcomes after immune checkpoint inhibitor exposure was identified in this review. This is the principal evidence gap and precludes CRC-specific estimates of direct gonadal risk.	Research gap identified in the present review.
Indirect CRC treatment-pathway and local-treatment evidence	Directly relevant to counsenoling because immune checkpoint blockade may modify exposure to established reproductive harms. In localized dMMR/MSI-H colon cancer, neoadjuvant or perioperative strategies remain trial-, pathway- and jurisdiction-dependent. In locally advanced dMMR/MSI-H rectal cancer, organ-preservation pathways may allow selected complete responders to avoid pelvic radiotherapy and radical surgery. When local treatment remains necessary, pelvic radiotherapy and rectal surgery can affect ovarian and uterine function, pelvic anatomy, sexual function and pregnancy feasibility. This evidence does not establish direct ovarian safety of ICIs.	CRC trials, clinical guidelines and survivorship literature [7,8,9,18,19,20,21,22,23,24,25,26,27,28].
Non-CRC human reproductive evidence	Extrapolative evidence from melanoma, renal cell carcinoma and mixed solid-tumor cohorts. Human data are sparse and heterogeneous, with limited observations on menstrual function, ovarian reserve markers and gonadal function; their generalizability to CRC regimens and treatment contexts is uncertain.	Systematic reviews, tumor-specific reviews and mixed-tumor cohorts [29,30,31,32,33,34].
Preclinical evidence	Provides biological plausibility for direct gonadal effects, including intra-ovarian immune activation, follicular loss and impaired oocyte competence after checkpoint blockade. These findings cannot be used to estimate infertility risk in women with CRC.	Murine and mechanistic models [37,38].
Endocrine toxicity evidence	Established and clinically actionable. Thyroid dysfunction, hypophysitis and pituitary dysfunction may cause amenorrhoea, anovulation or central hypogonadism independently of ovarian reserve and should be actively assessed during reproductive follow-up.	Endocrine toxicity cohorts and guidance [35,36].
Pregnancy and lactation safety evidence	Mechanistic and pharmacovigilance data support a conservative approach because PD-1/PD-L1 and CTLA-4 pathways contribute to maternal-fetal immune tolerance. Human evidence remains limited, heterogeneous and non-CRC-specific; it does not provide precise estimates of pregnancy risk after ICI exposure.	Systematic reviews and pharmacovigilance evidence [29,46,47].
Fertility-preservation guidance and clinical implementation	Provides the established basis for early counseling, specialist referral and consideration of oocyte, embryo or ovarian tissue cryopreservation according to patient preference, baseline reproductive vulnerability and oncologic timing. These recommendations guide implementation but do not establish ICI-specific gonadotoxicity.	ASCO, ESMO and ESHRE guidance; counseling, ovarian-reserve and fertility-preservation literature [10,11,12,48,49,50,51,52,53,54,55,56].
Regulatory product information	Provides agent- and jurisdiction-specific operational guidance on contraception and breastfeeding during treatment and after the last dose. These intervals should not be interpreted as direct evidence of ovarian toxicity or as universally applicable outside the relevant regulatory jurisdiction.	FDA prescribing information for pembrolizumab, nivolumab, ipilimumab and dostarlimab [41,42,43,44].

Abbreviations: AFC, antral follicle count; AMH, anti-Müllerian hormone; CRC, colorectal cancer; dMMR, deficient mismatch repair; ICI, immune checkpoint inhibitor; MSI-H, microsatellite instability-high.

**Table 2:** Representative clinical scenarios in reproductive-age women with colorectal cancer receiving or considered for immunotherapy and related counseling priorities.

Clinical Scenario	Main Reproductive Issue	Why Immunotherapy Matters (Clinical Context)	Counseling Priority
Localized dMMR/MSI-H colon cancer considered for neoadjuvant or perioperative immunotherapy	Need to decide whether fertility preservation can be discussed before immune-based treatment or surgery without compromising oncologic timing.	Investigational, trial-based or jurisdiction-dependent setting: immunotherapy may alter treatment sequence, but routine use remains pathway-dependent [21,23,24,25]. Trial protocols may also specify contraception requirements and pregnancy deferral.	Early documentation of reproductive goals; AMH/AFC and fertility history if relevant; rapid REI referral if a 10–14-day stimulation window is acceptable [10,11,12].
Locally advanced dMMR/MSI-H rectal cancer	High potential burden from pelvic radiotherapy, radical rectal surgery, altered pelvic anatomy, sexual dysfunction and pregnancy feasibility concerns [7,8,9,18,19].	Guideline-supported setting: PD-1 blockade may support complete clinical response and organ preservation in selected patients, potentially reducing pelvic treatment burden [18,19,22,26,27,28].	Counseling should cover fertility preservation, pelvic and sexual function, pregnancy feasibility, nonoperative management, surveillance, and contingency plans if response is incomplete.
Metastatic dMMR/MSI-H CRC treated with PD-1-based therapy, with or without CTLA-4 blockade	Long treatment horizon, uncertain conception timing, ongoing contraception, endocrine toxicity, possible treatment-free intervals after sustained disease control and future pregnancy questions in durable responders.	Standard-of-care setting: immunotherapy can provide durable disease control in selected patients [14,15,16,17,20]. Treatment interruption may occasionally create a treatment-free interval, but pregnancy remains contraindicated during active exposure.	Contraception, agent-specific washout, endocrine follow-up, ovarian reserve assessment when relevant, and individualized fertility preservation only if oncologically feasible.
Young patient with dMMR/MSI-H CRC and suspected or confirmed Lynch syndrome	Fertility preservation may intersect with hereditary cancer counseling and possible future PGT-M.	Molecular testing affects both systemic treatment strategy and reproductive planning [57,58].	Genetics referral; discussion of oocyte versus embryo cryopreservation; clarification that PGT-M requires embryo creation and a validated familial-variant testing pathway [58].
Patient developing endocrine irAEs during ICI therapy	Amenorrhea, anovulation or infertility may reflect thyroiditis, hypophysitis or central hypogonadism rather than primary ovarian failure [35,36].	Endocrine toxicity is an established complication of checkpoint blockade and is more actionable than unproven direct ovarian injury [35,36].	Low threshold for thyroid and pituitary evaluation, hormone replacement when indicated, and distinction between ovarian reserve loss and endocrine-mediated reproductive dysfunction [35,36].

**Table 3:** FDA-label-informed reproductive safety counseling for selected immune checkpoint inhibitors relevant to colorectal cancer.

Agent	Typical CRC Relevance	Post-Treatment Contraception Interval	Breastfeeding Counseling
Pembrolizumab (PD-1)	dMMR/MSI-H metastatic CRC; selected perioperative or neoadjuvant pathways depending on jurisdiction, guideline context and protocol.	Use effective contraception during treatment and for 4 months after the last dose.	Do not breastfeed during treatment and for 4 months after the last dose.
Nivolumab (PD-1)	dMMR/MSI-H metastatic CRC, including combinations with ipilimumab; selected neoadjuvant or perioperative studies.	Use effective contraception during treatment and for 5 months after the last dose.	Do not breastfeed during treatment and for 5 months after the last dose.
Ipilimumab (CTLA-4)	Combination regimens with nivolumab in metastatic or neoadjuvant dMMR/MSI-H CRC contexts.	Use effective contraception during treatment and for 3 months after the last dose.	Do not breastfeed during treatment and for 3 months after the last dose.
Dostarlimab (PD-1)	Locally advanced dMMR/MSI-H rectal cancer and selected dMMR solid tumor pathways according to regulatory status and institutional practice.	Use effective contraception during treatment and for 4 months after the last dose.	Do not breastfeed during treatment and for 4 months after the last dose.

Note: intervals summarize U.S. FDA prescribing information available at the search cutoff and should be checked against current product information in the relevant jurisdiction before individual counseling. In combination regimens, the longest relevant interval should generally be applied [41,42,43,44].

## Data Availability

No new data were created or analyzed in this study. Data sharing is not applicable to this article.

## Abbreviations

AFC	Antral Follicle Count
AMH	Anti-Müllerian Hormone
ASCO	American Society of Clinical Oncology
BRAF	B-Raf Proto-Oncogene, Serine/Threonine Kinase
CRC	Colorectal Cancer
CTLA-4	Cytotoxic T-Lymphocyte-Associated Protein 4
dMMR	Deficient Mismatch Repair
EOCRC	Early-Onset Colorectal Cancer
ESHRE	European Society of Human Reproduction and Embryology
ESMO	European Society for Medical Oncology
FDA	Food and Drug Administration
FSH	Follicle-Stimulating Hormone
GnRH	Gonadotropin-Releasing Hormone
HER2	Human Epidermal Growth Factor Receptor 2
ICI	Immune Checkpoint Inhibitor
irAE	Immune-Related Adverse Event
IVM	In Vitro Maturation
LH	Luteinizing Hormone
MEDLINE	Medical Literature Analysis and Retrieval System Online
MMR	Mismatch Repair
MRI	Magnetic Resonance Imaging
MSI	Microsatellite Instability
MSI-H	Microsatellite Instability-High
MSS	Microsatellite Stable
PD-1	Programmed Cell Death Protein 1
PD-L1	Programmed Death-Ligand 1
PGT-M	Preimplantation Genetic Testing for Monogenic Disorders
pMMR	Proficient Mismatch Repair
RAS	Rat Sarcoma Viral Oncogene Homolog
REI	Reproductive Endocrinology and Infertility
SANRA	Scale for the Assessment of Narrative Review Articles
